# Structural covariance of the ventral visual stream predicts posttraumatic intrusion and nightmare symptoms: a multivariate data fusion analysis

**DOI:** 10.1038/s41398-022-02085-8

**Published:** 2022-08-08

**Authors:** Nathaniel G. Harnett, Katherine E. Finegold, Lauren A. M. Lebois, Sanne J. H. van Rooij, Timothy D. Ely, Vishnu P. Murty, Tanja Jovanovic, Steven E. Bruce, Stacey L. House, Francesca L. Beaudoin, Xinming An, Donglin Zeng, Thomas C. Neylan, Gari D. Clifford, Sarah D. Linnstaedt, Laura T. Germine, Kenneth A. Bollen, Scott L. Rauch, John P. Haran, Alan B. Storrow, Christopher Lewandowski, Paul I. Musey, Phyllis L. Hendry, Sophia Sheikh, Christopher W. Jones, Brittany E. Punches, Michael C. Kurz, Robert A. Swor, Lauren A. Hudak, Jose L. Pascual, Mark J. Seamon, Erica Harris, Anna M. Chang, Claire Pearson, David A. Peak, Robert M. Domeier, Niels K. Rathlev, Brian J. O’Neil, Paulina Sergot, Leon D. Sanchez, Mark W. Miller, Robert H. Pietrzak, Jutta Joormann, Deanna M. Barch, Diego A. Pizzagalli, John F. Sheridan, Steven E. Harte, James M. Elliott, Ronald C. Kessler, Karestan C. Koenen, Samuel A. McLean, Lisa D. Nickerson, Kerry J. Ressler, Jennifer S. Stevens

**Affiliations:** 1 Division of Depression and Anxiety, McLean Hospital, Belmont, MA USA; 2 Department of Psychiatry, Harvard Medical School, Boston, MA USA; 3 Department of Psychiatry and Behavioral Sciences, Emory University School of Medicine, Atlanta, GA USA; 4 Department of Psychology, Temple University, Philadelphia, PA USA; 5 Department of Psychiatry and Behavioral Neurosciences, Wayne State University, Detroit, MI USA; 6 Department of Psychological Sciences, University of Missouri - St. Louis, St. Louis, MO USA; 7 Department of Emergency Medicine, Washington University School of Medicine, St. Louis, MO USA; 8 Department of Emergency Medicine & Department of Health Services, Policy, and Practice, The Alpert Medical School of Brown University, Rhode Island Hospital and The Miriam Hospital, Providence, RI USA; 9 Institute for Trauma Recovery, Department of Anesthesiology, University of North Carolina at Chapel Hill, Chapel Hill, NC USA; 10 Department of Biostatistics, Gillings School of Global Public Health, University of North Carolina, Chapel Hill, NC USA; 11 Departments of Psychiatry and Neurology, University of California San Francisco, San Francisco, CA USA; 12 Department of Biomedical Informatics, Emory University School of Medicine, Atlanta, GA USA; 13 Department of Biomedical Engineering, Georgia Institute of Technology and Emory University, Atlanta, GA USA; 14 Institute for Technology in Psychiatry, McLean Hospital, Belmont, MA USA; 15 The Many Brains Project, Belmont, MA USA; 16 Department of Psychology and Neuroscience & Department of Sociology, University of North Carolina at Chapel Hill, Chapel Hill, NC USA; 17 Department of Psychiatry, McLean Hospital, Belmont, MA USA; 18 Department of Emergency Medicine, University of Massachusetts Chan Medical School, Worcester, MA USA; 19 Department of Emergency Medicine, Vanderbilt University Medical Center, Nashville, TN USA; 20 Department of Emergency Medicine, Henry Ford Health System, Detroit, MI USA; 21 Department of Emergency Medicine, Indiana University School of Medicine, Indianapolis, IN USA; 22 Department of Emergency Medicine, University of Florida College of Medicine-Jacksonville, Jacksonville, FL USA; 23 Department of Emergency Medicine, Cooper Medical School of Rowan University, Camden, NJ USA; 24 Department of Emergency Medicine, Ohio State University College of Medicine, Columbus, OH USA; 25 Ohio State University College of Nursing, Columbus, OH USA; 26 Department of Emergency Medicine, University of Alabama School of Medicine, Birmingham, AL USA; 27 Department of Surgery, Division of Acute Care Surgery, University of Alabama School of Medicine, Birmingham, AL USA; 28 Center for Injury Science, University of Alabama at Birmingham, Birmingham, AL USA; 29 Department of Emergency Medicine, Oakland University William Beaumont School of Medicine, Rochester, MI USA; 30 Department of Emergency Medicine, Emory University School of Medicine, Atlanta, GA USA; 31 Department of Surgery, Department of Neurosurgery, University of Pennsylvania, Philadelphia, PA USA; 32 Perelman School of Medicine, University of Pennsylvania, Philadelphia, PA USA; 33 Department of Surgery, Division of Traumatology, Surgical Critical Care and Emergency Surgery, University of Pennsylvania, Philadelphia, PA USA; 34 Einstein Medical Center, Philadelphia, PA USA; 35 Department of Emergency Medicine, Jefferson University Hospitals, Philadelphia, PA USA; 36 Department of Emergency Medicine, Wayne State University, Ascension St. John Hospital, Detroit, MI USA; 37 Department of Emergency Medicine, Massachusetts General Hospital, Boston, MA USA; 38 Department of Emergency Medicine, Saint Joseph Mercy Hospital, Ypsilanti, MI USA; 39 Department of Emergency Medicine, University of Massachusetts Medical School-Baystate, Springfield, MA USA; 40 Department of Emergency Medicine, Wayne State University, Detroit Receiving Hospital, Detroit, MI USA; 41 Department of Emergency Medicine, McGovern Medical School, University of Texas Health, Houston, TX USA; 42 Department of Emergency Medicine, Brigham and Women’s Hospital, Boston, MA USA; 43 Department of Emergency Medicine, Harvard Medical School, Boston, MA USA; 44 National Center for PTSD, Behavioral Science Division, VA Boston Healthcare System, Boston, MA USA; 45 Department of Psychiatry, Boston University School of Medicine, Boston, MA USA; 46 National Center for PTSD, Clinical Neurosciences Division, VA Connecticut Healthcare System, West Haven, CT USA; 47 Department of Psychiatry, Yale School of Medicine, New Haven, CT USA; 48 Department of Psychology, Yale University, New Haven, CT USA; 49 Department of Psychological & Brain Sciences, Washington University in St. Louis, St. Louis, MO USA; 50 Division of Biosciences, Ohio State University College of Dentistry, Columbus, OH USA; 51 Institute for Behavioral Medicine Research, OSU Wexner Medical Center, Columbus, OH USA; 52 Department of Anesthesiology, University of Michigan Medical School, Ann Arbor, MI USA; 53 Department of Internal Medicine-Rheumatology, University of Michigan Medical School, Ann Arbor, MI USA; 54 Kolling Institute, University of Sydney, St Leonards, New South Wales Australia; 55 Faculty of Medicine and Health, University of Sydney, Northern Sydney Local Health District, New South Wales, Australia; 56 Physical Therapy & Human Movement Sciences, Feinberg School of Medicine, Northwestern University, Chicago, IL USA; 57 Department of Health Care Policy, Harvard Medical School, Boston, MA USA; 58 Department of Epidemiology, Harvard T.H. Chan School of Public Health, Harvard University, Boston, MA USA; 59 Department of Emergency Medicine, University of North Carolina at Chapel Hill, Chapel Hill, NC USA; 60 Institute for Trauma Recovery, Department of Psychiatry, University of North Carolina at Chapel Hill, Chapel Hill, NC USA; 61 McLean Imaging Center, McLean Hospital, Belmont, MA USA

**Keywords:** Human behaviour, Prognostic markers, Psychiatric disorders

## Abstract

Visual components of trauma memories are often vividly re-experienced by survivors with deleterious consequences for normal function. Neuroimaging research on trauma has primarily focused on threat-processing circuitry as core to trauma-related dysfunction. Conversely, limited attention has been given to visual circuitry which may be particularly relevant to posttraumatic stress disorder (PTSD). Prior work suggests that the ventral visual stream is directly related to the cognitive and affective disturbances observed in PTSD and may be predictive of later symptom expression. The present study used multimodal magnetic resonance imaging data (*n* = 278) collected two weeks after trauma exposure from the AURORA study, a longitudinal, multisite investigation of adverse posttraumatic neuropsychiatric sequelae. Indices of gray and white matter were combined using data fusion to identify a structural covariance network (SCN) of the ventral visual stream 2 weeks after trauma. Participant’s loadings on the SCN were positively associated with both intrusion symptoms and intensity of nightmares. Further, SCN loadings moderated connectivity between a previously observed amygdala-hippocampal functional covariance network and the inferior temporal gyrus. Follow-up MRI data at 6 months showed an inverse relationship between SCN loadings and negative alterations in cognition in mood. Further, individuals who showed decreased strength of the SCN between 2 weeks and 6 months had generally higher PTSD symptom severity over time. The present findings highlight a role for structural integrity of the ventral visual stream in the development of PTSD. The ventral visual stream may be particularly important for the consolidation or retrieval of trauma memories and may contribute to efficient reactivation of visual components of the trauma memory, thereby exacerbating PTSD symptoms. Potentially chronic engagement of the network may lead to reduced structural integrity which becomes a risk factor for lasting PTSD symptoms.

## Introduction

Traumatic memories in posttraumatic stress disorder (PTSD) can spontaneously intrude into a victim’s thoughts, contributing to re-experiencing of the event, or prompting hypervigilance to potential cues that signal danger [1]. Though often assumed to reflect alterations in core threat neurocircuitry, the process of encoding, recalling, and directing attention to visual information in PTSD may also rely on visual circuitry that has been generally understudied [2]. Vividness of imagined scenes in those with PTSD has long been known to be positively related to frequency of posttraumatic flashbacks and nightmares, underscoring the potential clinical relevance of visual processing in the disorder [3]. Putatively aberrant neurobiology of visuo-affective circuitry may thus help to clarify PTSD-related behavioral changes such as threat vigilance, flashbacks, nightmares, and intrusive thoughts [4–8].

The ventral visual stream may be particularly important for PTSD given its role in object recognition and categorization, and its interconnections with canonical threat-related regions such as the amygdala for processing of threat-relevant stimuli [9–14]. Early visual cortex regions (V1 and V2), the posterior/anterior inferior temporal lobe, and superior temporal sulcus are thought to be canonical regions of the ventral visual stream with known projections to regions such as the amygdala, medial temporal lobe, and striatum [15]. Sensory information is thought to move from primary visual cortices along the pathway towards more anterior components wherein more complex and higher-order features tend to be represented. The ventral visual stream has also previously been proposed to be critical for the development of the prefrontal cortex (PFC) and executive functions including attention regulation [16]. Both animal model and human neuroimaging research also demonstrate that stress exposure modulates activity in neural regions of visual processing [17, 18]. Extant literature therefore suggests visual circuitry may be involved in stress-modulated threat responses, but limited work in PTSD has directly investigated potential disorder-relevant circuitry variability.

Consideration of visual circuitry may further the development of functional and structural magnetic resonance imaging (MRI)-based neural signatures of PTSD susceptibility. Prior functional MRI (fMRI) studies suggest amygdala reactivity to threat and connectivity to the PFC either before or relatively early after trauma is associated with greater risk to developing later PTSD symptoms [19–22]. However, ventral visual areas also show increased reactivity to threat in chronic PTSD [23] and amygdala to visual cortex connectivity is related to later PTSD symptoms above and beyond current PTSD symptoms [22]. Moreover, structural MRI research suggests hippocampal volume and uncinate fasciculus microstructure are predictive of PTSD, though these findings are mixed [24–30]. Other white matter imaging research, however, has found that microstructure of visual association tracts such as the inferior fronto-occipital fasciculus and inferior longitudinal fasciculus in the early aftermath of trauma is related to PTSD development [31]. Thus while neurobiology of threat circuitry is important, integration of functional and structural correlates of visual circuitry may enhance our understanding of the neurobiology of PTSD susceptibility.

Multimodal data fusion approaches to brain imaging data may be better suited to uncovering affective visual neurocircuitry relevant to PTSD than previously used unimodal approaches. Multivariate analyses allow for simultaneous integration of information across multiple imaging modalities to identify associations with PTSD symptoms, thus increasing the overall power of MRI data to identify PTSD-susceptible phenotypes. Prior work observed multimodal patterns of fMRI and positron emission tomography (PET) activation distributed across threat neurocircuitry that also included thalamus, extrastriatal, and primary visual cortex differentially associated with PTSD and traumatic brain injury-related dysfunction [32]. Similarly, we have previously observed an association between the strength of a multimodal ventral visual stream structural covariance network and early PTSD symptoms after trauma in a distinct, smaller trauma cohort [33]. Together, the prior findings highlight the increased power and utility of multimodal neuroimaging for dissecting trauma-related disorder phenotypes and the potential role of both threat and visual neurocircuitry. Whether changes in structure of the ventral visual stream over time, or potential relationships to brain function, are related to PTSD symptoms remains unclear.

In the present study, we utilized data from the large, prospective, longitudinal Advancing Understanding of RecOvery afteR trauma (AURORA) study to investigate multimodal MRI markers of PTSD. We first investigated if a previously observed structural covariance network (SCN) of the ventral visual stream (VVS) would be replicated in a separate, multisite, and demographically heterogenous dataset. We hypothesized that individual variability in the strength of the SCN would be positively related to acute PTSD symptoms. We also examined the relationship between variability in the SCN loadings and post-trauma nightmare symptoms as an index of vision-related posttraumatic disturbance. In addition, we investigated if the VVS SCN would be related to threat-related neural reactivity or connectivity patterns previously shown to be related to PTSD susceptibility in emergency department cohorts. Finally, we evaluated if changes in SCN loadings were associated with changes in PTSD symptoms over time. The present findings provide a robust characterization of a multimodal structural covariance network of the ventral visual stream in recent trauma victims and establishes a new framework for potential affective-visual circuitry that may be critical to understanding PTSD susceptibility.

## Methods

Data from the present analyses were obtained as part of the AURORA Study, a multisite longitudinal study of adverse posttraumatic neuropsychiatric sequelae. Details of the larger AURORA project are described in the parent and recent reports [22, 34–36]. Briefly, trauma-exposed participants were recruited from Emergency Departments (ED) from across the United States. Trauma was defined as a medical accident requiring admission to the ED, and participants who experienced events such as a motor vehicle collision, high fall (>10 feet), physical assault, sexual assault, or mass casualty incidents were automatically included in the study. Other trauma exposures were also qualifying if: (a) the individual responded to a screener question that they experienced the exposure as involving actual or threatened serious injury, sexual violence, or death, either by direct exposure, witnessing, or learning about the trauma and (b) the research assistant agreed that the exposure was a plausible qualifying event. Trauma was a necessary inclusion criterion for the present study, and no participants without trauma were included. Frequency of the broad class of trauma exposures endorsed by participants are included in Table S1. Data were initially intended to be collected from 439 participants recruited between 09/25/2017 and 07/31/2020. Of the recruited participants, no MRI data were collected from 53 participants. Further, the present multimodal data fusion analysis required participants to have completed both anatomical T1-weighted and diffusion weighted imaging (DWI). Thus, we excluded participants without either data type or whose data did not pass quality control procedures (described below). In total, 278 participants were included in the multimodal data fusion analyses. A subset of individuals (*n* = 83) also had usable MRI data from a follow-up imaging session 6 months after trauma exposure. All participants gave written informed consent as approved by each study site’s Institutional Review Board.

### Demographic and psychometric data collection

Initial participant demographic and psychometric data were collected after admission to the ED (Table 1). PTSD symptoms were assessed using the PTSD Checklist for DSM-5 (PCL-5) [37], a 20-item self-report questionnaire on symptom expression and severity. Participants’ PTSD symptoms were assessed within the ED (i.e., past 30 days prior to trauma), 2 weeks, 8 weeks, 3 months, and 6 months after trauma exposure. For the present analyses, we focused on prior (30 days prior to ED), 2-week and 6-month PTSD symptoms post ED given our a priori interests and to limit the number of potential comparisons. The 2-week assessment queried participant symptoms in the past 14 days while the 6-month assessment queried participant symptoms in the past 30 days. Participants’ depression symptoms were also assessed using the Patient-Reported Outcomes Measurement Information System (PROMIS) Depression instrument from the PROMIS short form 8b [38]. T-scores were derived from total responses to eight items scored on a Likert scale from 1 (never) to 5 (always). Participants’ prior life trauma was assessed using the Life Events Checklist version 5 (LEC-5) [39]. The checklist assessed prior exposure to traumatic events such as natural disasters, accidents, assaults, etc. that (a) happened directly to the participant, (b) were witnessed by the participant, (c) the participant learned happened to someone close to them, or (d) the participant was exposed to details of it due to their occupation. Responses to all questions were summed to derive a prior trauma index and participants who did not respond to all questions were coded as missing. Participants’ symptoms of nightmares were assessed two weeks after trauma using the Clinician Administered PTSD Scale IV [40, 41]. Specifically, participants were asked, in the past two weeks, (1) how often they had unpleasant dreams and (2) how much distress or discomfort did their unpleasant dreams cause? Participants scored each question on a scale from 0 to 4. Nightmare frequency was defined as participant responses to question one. Nightmare intensity was defined as participant responses to question two. Nightmare severity was defined as the sum of responses to questions one and two.

**Table 1:** Participant demographics.

Characteristic	Mean (SD) or *n* (%)
Age	33.99 (12.83)
Sex assigned at birth, male/female	102 (36.69%)/176 (63.31%)
Race/ethnicity	
Hispanic	45 (16.19%)
White	98 (35.25%)
Black	122 (43.89%)
Other	11 (3.96%)
Missing	2 (0.72%)
Highest grade level	
12th grade or less (No diploma)	12 (4.32%)
High school graduate or GED	77 (27.70%)
Some college (No degree)	90 (32.37%)
Associates degree (Occupational/Vocational or Academic program)	31 (11.15%)
Bachelor's degree	50 (17.99%)
Graduate degree (Master's, Professional, or Doctoral)	18 (6.48%)
Income level	
<$19,000	64 (23.02%)
$19,001–$35,000	86 (30.94%)
$35,001–$50,000	37 (12.31%)
$50,001–$75,000	27 (9.71%)
$75,001–$100,000	15 (5.40%)
>$100,000	21 (7.55%)
Missing	28 (10%)
*Clinical characteristics*	
PCL-5 scores	
(30 days pre-ED) (*n* = 201)	30.76 (15.88)
2-week (*n* = 244)	30.50 (17.14)
6-month (*n* = 199)	22.17 (17.49)
PROMIS depression	
Prior (30 days pre-ED) (*n* = 277)	49.87 (10.71)
2-week (*n* = 255)	55.63 (9.83)
6-month (*n* = 197)	52.49 (10.47)
LEC-5 scores (*n* = 220)	10.49 (10.13)

### Magnetic resonance imaging

#### General procedures

MRI data were collected across five sites with harmonized acquisition parameters (Table S2). In the present analyses, we utilized participants’ T1-weighted, DWI, task-fMRI during a threat reactivity task, and resting-state fMRI data. Results included in this manuscript come from preprocessing performed using FMRIPREP version stable 1.2.2, a Nipype-based tool [42, 43]. Details of the pipeline are described in the supplemental material. Modality and feature-specific processing are described below. A schematic of the overall processing and analytic steps are provided in the supplement (Figure S1).

### Voxel-based morphometry and surface-based morphology

T1-weighted MRI data were visually inspected and assessed using MRIQC [44]. We excluded participants based on coefficient of joint variation (CJV), a measure of motion and voxel non-uniformity, by a value of >2 SD (*n* = 23). Voxel-based morphometry (VBM) and cortical surface features were generated as in our prior reports [28, 33]. VBM was completed using standard FSL routines (i.e., FSLVBM) of the participants’ anatomical scan [45–47]. Normalized and modulated gray matter volume (GMV) maps were smoothed using a 4 mm full-width at half-maximum (FWHM) Gaussian Kernel and re-masked by a participant-derived gray matter mask. The VBM results were visually inspected and participants with poor segmentation were omitted (*n* = 24) from the multimodal analysis. Cortical surface maps were reconstructed through FreeSurfer within the FMRIPREP framework [42, 43]. Individual participant maps of cortical thickness (CT) and pial surface area (PSA) were resampled into the fsaverage space and smoothed at a range of 10 mm FWHM.

### Diffusion tensor imaging (DTI)

DTI of the white matter skeleton was completed to derive metrics of white matter microstructure as described in our prior reports [28, 33]. Briefly, the processing pipeline was developed according to the recommendations of the ENIGMA consortium (http://enigma.ini.usc.edu/protocols/dti-protocols/). To ensure quality data, we calculated metrics of temporal signal-to-noise ratio and outlier maximum voxel intensity as in a prior report [47]. Participants who demonstrated both (a) TSNR values lower than 4.88 and (b) maximum voxel intensities >5000 were removed from analyses to retain the maximum number of participants while removing low-quality data. Motion and eddy current effects in diffusion data were reduced using the ‘eddy’ subroutine in the FMRIB Software Library (FSL) [47–49] and susceptibility effects were corrected using nonlinear warping of the diffusion data to the participant’s anatomical data [50]. Diffusion data were fit with a tensor model and Tract-Based Spatial Statistics (TBSS) processing was implemented following the ENIGMA-DTI working group processing standards to generate fractional anisotropy (FA), mean diffusivity (MD), and mode of the diffusion tensor (MO) skeletal maps [51, 52].

### Task-fMRI

To index neural reactivity to threat, participants completed an emotional reactivity task designed to probe reactivity to social threat cues via passive viewing of fearful and neutral facial expressions. The task is described in prior work [19, 23, 36]. Faces from the Ekman faces library were presented in a block design with 8 different faces of the same emotion (fear vs. neutral) within an 8 s block. Individual faces were presented for 500 ms each with a 500 ms interstimulus interval. Participants were given three 10 s rest periods evenly distributed throughout the task. The 5 min task included 15 fear blocks and 15 neutral blocks presented in a pseudorandom order. The order was counterbalanced across participants. The 1st-level models of these data were completed in SPM12 after denoising with ICA-AROMA as part of the FMRIPREP pipeline, which has been shown to handle motion artifacts in a robust, data-driven fashion that performs equal to and in some cases better than standard scrubbing or censoring procedures at the individual participant level [53–55]. Task blocks were modeled with separate boxcar functions representing the onset and 8000 ms duration of each block, convolved with a canonical hemodynamic response function. Separate regressors for white matter, cerebrospinal fluid, and global signal were included to account for any remaining motion/physiological noise following ICA. Group-level statistical modeling was completed in AFNI on the 1st level contrasts of fearful > neutral face contrast.

### Resting-state fMRI

Resting state fMRI data were further processed within the Analysis for Functional NeuroImages (AFNI) program 3dTproject following denoising with ICA-AROMA to perform linear detrending, censoring of non-steady state volumes identified by FMRIPREP, bandpass filtering (0.01–0.1 Hz), and regression of white matter, corticospinal fluid, and global signal to account for potential physiological noise. Group ICA via MELODIC was completed as described in our prior report [22]. Automatic dimensionality reduction selected 77 independent components. We focused the initial analyses on two RSNs based on our a priori hypotheses and findings from an earlier report: the default mode network and an amygdala-hippocampal functional covariance network [22]. Spatial maps of selected RSNs for analysis are depicted in the supplement (Figure S2). Exploratory analyses of additional spatial maps that covered visual and “arousal” regions seen in our prior work were also completed (*n* = 6) [33]. Dual regression was performed to derive individual participant spatial maps for each component as described previously [22, 56, 57].

### Linked independent component analysis

Linked independent component analysis (LICA) was completed using FSL and MATLAB to perform multimodal data fusion [58, 59]. LICA leverages joint information reflected in the MR modalities to identify correlated patterns of structural and diffusion spatial variation that are linked together through a subjects loading matrix reflecting the strength of the component pattern for each participant. Each identified component is thus composed of two parts: (1) a spatial map, for each modality, that describes regional variability that is related to variability to all other modalities and (2) participant loadings that describe an individual participant’s relative strength of the component relative to all other participants. The FA, MD, MO, GMV, CT, and PSA maps from the 2-week MRI scan were used as spatial features. LICA was completed at a high dimensionality (L = 119, the maximum estimated within LICA) to better separate signal and noise (i.e., participant-dominated and motion) components [33, 59–61]. We specifically focused on identifying a component reflecting the ventral visual stream and thus only selected a single component for analyses to limit multiple comparisons. A subset (*n* = 83) of participants had usable, longitudinal multimodal MRI data from an identical imaging session completed 6 months after trauma exposure. Components from the LICA performed with 2-week MRI data were projected to the 6-month data via multivariate spatial regression similar to the first stage of dual regression, for each modality separately [61] to derive a set of subject loadings for each modality. The loadings were then averaged together to generate an estimated 6-month component loading.

### Statistical analyses

Statistical analyses were completed using IBM SPSS version 24, the JASP Statistical Package (https://jasp-stats.org/), and the Analysis of Functional NeuroImages (AFNI) software package [62]. We completed multiple linear regressions to evaluate if SCN loadings were associated with acute (i.e., 2-week) PTSD symptoms. Regressions included dummy-coded scanner and sex assigned at birth covariates as well as continuous covariates for age at enrollment, pre-trauma PTSD symptoms reported in the ED, and total score on the LEC-5. Linear and quadratic predictors of 2-week PTSD from the PCL-5 were included. The model was chosen to harmonize with our previously reported approach [33]. Inclusion of the prior PTSD symptoms and LEC-5 scores reduced the sample size for analyses and thus we also completed the regressions removing these covariates. In the prior report, we observed a positive relationship between VVS SCN loadings and PTSD symptoms, with some specificity for re-experiencing symptoms and thus we anticipated a similar effect in the present analyses. Given our a priori hypothesis on the association between 2-week SCN loadings and PCL-5 scores, we set a nominal *p*-value of 0.05 with one-tailed testing for the model with a Bonferroni–Holm correction to account for the multiple comparisons (models with and without covariates). Similar models were completed for estimated 6-month SCN loadings, however, the quadratic term was removed given findings from the previous models (described in the “Results”). Given the lack of a directional hypothesis for these sets of tests, we set a nominal *p*-value 0.05 with two-tailed testing for the models and a Bonferroni–Holm correction was applied for each set (i.e., with and without covariates) of models. Significant associations with total PTSD symptom severity in the above models were followed-up with identical models substituting PTSD subscale scores to understand the domains driving the effect. For models with a significant subscale association with SCN loadings, we compared standardized regression coefficients between the significant model and all other models using a Z-test [63]. We further completed repeated-measures analysis of covariance (RM-ANCOVA) to assess the effect of changes in component loadings on PCL-5 scores (dependent variable) with a within-subject’s factor for time (2 weeks and 6 months), a continuous measure of change in VVS SCN loadings (6-month minus 2-week), and covariates for age, sex assigned at birth, and scanner site. We also completed multiple regressions to assess the relationship between 2-week component loadings and Clinician Administered PTSD Scale for DSM-IV (CAPS-IV) nightmare frequency, intensity, and severity (3 separate models) while covarying for scanner site, age, and sex assigned at birth. A Bonferroni–Holm correction was applied to the models of nightmare symptoms to account for multiple comparisons. We present only a priori outcomes of interest from regression models in the results, but full outputs are available in the supplement. Voxelwise group-level models of the resting state fMRI data were completed using 3dttest++ in AFNI for the default mode and amygdala-hippocampal functional covariance networks (two separate models). Models included a continuous regressor of loadings from the ventral visual stream SCN as well as covariates for scanner, age, and sex assigned at birth. A gray matter mask that included subcortical areas and the cerebellum was applied to the data. Permutation-based cluster correction (i.e., the -clustsim option in 3dttest++) was used to determine the corresponding voxel extent *k* needed at a cluster forming threshold of *p* = 0.005 to maintain *α* = 0.05 (10,000 iterations). Cluster-corrected results were considered significant at a corrected *p* < 0.025 to account for the two networks assessed. A separate voxelwise comparison was completed for the threat reactivity task. Given that the present study used pooled, multisite data, we completed supplementary assessments of data quality between each site and these analyses are documented in the supplementary material (Figure S2).

## Results

### Participant demographics

Participant demographics are presented in Table 1. Correlations between the psychometric instruments are presented in Table 2. Indices of PTSD and depression were correlated at each timepoint, and these were in turn related to CAPS-IV nightmare frequency, intensity, and severity. Independent samples *t*-tests did not reveal a significant difference in PCL-5 scores between participants who did or did not provide 6-month MRI data either at 2 weeks [t(242) = 1.27, *p* = 0.207] or 6 months [t(197) = −0.21, *p* = 0.831]. Thus the present data come from a largely female, racially/ethnically diverse sample with varying degrees of posttraumatic dysfunction.

**Table 2:** Psychometric variable correlations.

Variables	PCL-5 (prior)	PCL-5 (2 weeks)	PCL-5 (6 months)	PROMIS depression (prior)	PROMIS depression (2 weeks)	PROMIS depression (6 months)	LEC-5 (total score)	Nightmare frequency	Nightmare intensity
1. PCL-5 (2 weeks)	0.524** (177)								
2. PCL-5 (6 months)	0.492** (145)	0.507** (185)							
3. PROMIS Depression (prior)	0.532** (200)	0.360** (243)	0.303** (198)						
4. PROMIS Depression (2 weeks)	0.446** (186)	0.757** (244)	0.429** (191)	0.524** (254)					
5. PROMIS Depression (6 months)	0.387** (144)	0.410** (184)	0.804** (193)	0.428** (196)	0.504** (190)				
6. LEC-5 (total score)	0.066 (158)	0.193** (203)	0.115 (177)	0.138* (219)	0.119 (213)	0.087 (174)			
7. Nightmare Frequency	0.325** (185)	0.487** (243)	0.330** (190)	0.191** (252)	0.383** (253)	0.220** (189)	−0.046 (212)		
8. Nightmare intensity	0.293** (185)	0.490** (243)	0.373** (190)	0.178** (252)	0.365** (253)	0.253** (189)	0.01 (212)	0.729** (253)	
9. Nightmare severity	0.333** (185)	0.525** (243)	0.375** (190)	0.198** (252)	0.402** (253)	0.252** (189)	−0.019 (212)	0.926** (253)	0.933** (253)

*p < 0.05, **p < 0.01, PCL-5 = PTSD Checklist for DSM-5; PROMIS = Patient-Reported Outcomes Measurement Information System. Data presented as *r*-value (*n*).

### Structural covariance of the ventral visual stream is associated with acute posttraumatic dysfunction

**Fig. 1:**
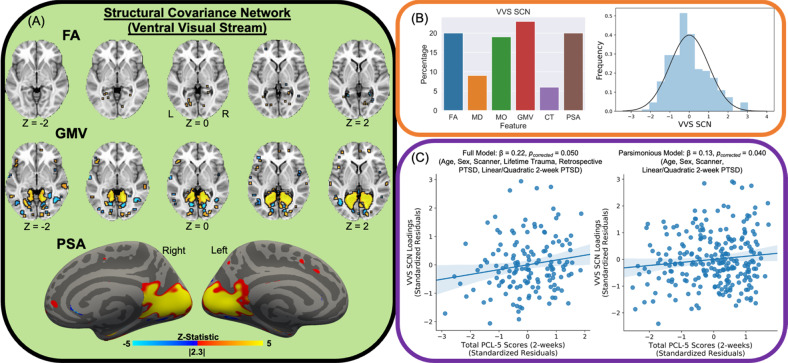
Structural covariance network of the ventral visual stream and acute PTSD severity. Linked independent components analysis (LICA) was completed on fractional anisotropy (FA), mean diffusivity (MD), mode of the diffusion tensor (MO), gray matter volume (GMV), cortical thickness (CT), and pial surface area (PSA) spatial maps to derive multimodal components. We observed a component that reflected a structural analog of the ventral visual stream derived from all participants (**A**). The component predominantly reflected FA, GMV, and PSA of the visual cortex, anterior temporal lobe, and the inferior fronto-occipital fasciculus (left) and the distribution of loadings across participants (right) (**B**). The component was related to total scores on the PTSD Checklist for DSM-5 (PCL-5) in both full (left) and parsimonious (right) models described in the “Methods” section (**C**). Brain figures represent the ventral visual stream structural covariance network obtained from LICA across all participants. Scatter plots are partial plots where dots represent the standardized residuals of individual participant loadings and PCL-5 scores. Lines represent the linear line of best fit and the shaded bar represents the 95% confidence interval.

LICA revealed a structural covariance network (SCN) of the ventral visual stream (VVS) associated with acute PTSD severity (PCL-5 scores) (Fig. 1; Table S3). The SCN primarily reflected fractional anisotropy (FA; 20%), gray matter volume (GMV; 23%), and pial surface area (PSA; 20%) of the inferior fronto-occipital fasciculus, visual cortex, and anterior temporal pole (i.e., bilateral medial aspects of the ventral visual stream; VVS). In line with our a priori hypothesis, loadings on the SCN were significantly linearly associated with PTSD symptoms at 2 weeks, with [t(133) = 1.98, *β* = 0.22, 95% CI = [0.02, 0.42], *p*_corrected_ = 0.05, one-tailed] and without [t(235) = 1.77, *β* = 0.13, 95% CI = [−0.01, 0.27], *p*_corrected_ = 0.04, one-tailed] the inclusion of covariates for prior trauma history (LEC-5 total score) and pre-trauma PTSD symptoms. Given the prior findings, we assessed if the associations were specific to individual PTSD symptom dimensions (indexed by PCL-5 subscale scores). Loadings on the SCN were significantly linearly associated with intrusion symptoms (e.g., flashbacks, re-experiencing, etc.) [t(133) = 2.16, *β* = 0.23, 95% CI = [0.01, 0.45], *p* = 0.033], but not avoidance, negative cognition/mood, or arousal symptoms (all *p* > 0.05). Comparison of *β*-values revealed the association between the SCN loadings and intrusion symptoms was significantly different compared to the association with avoidance (Z-value = 2.30, *p* = 0.022), negative alterations in cognition and mood (Z-value = 2.62, 0.009), and arousal (Z-value = 2.54, *p* = 0.011) subscales. We further completed supplementary analyses to determine if similar associations were observed between VVS SCN loadings and depression symptoms (using the PROMIS). We did not observe any linear or curvilinear associations between VVS SCN loadings and depressive symptoms with or without accounting for prior trauma or pre-trauma PTSD symptoms (Table S4). These data replicate prior observations of a positive association between VVS SCN loadings and re-experiencing symptoms in a smaller sample [33] and suggest structural covariance of the VVS is positively related to intrusive memories in the aftermath of traumatic stress.

**Fig. 2:**
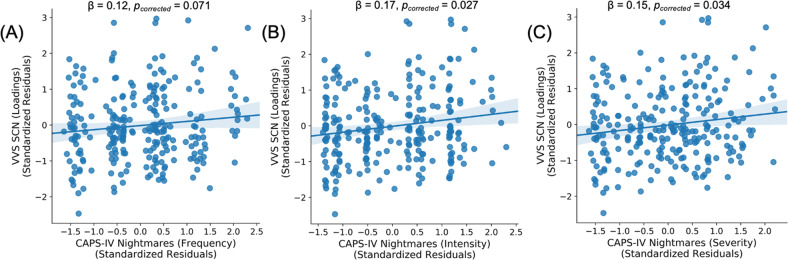
Ventral visual stream structural covariance strength is related to nightmare symptoms. Individual participant loadings on the VVS SCN identified through linked independent components analysis (LICA) varied positively with participants’ nightmare frequency (**A**), intensity (**B**), and severity (**C**) scores at 2 weeks post trauma. Scatter plots are partial plots where dots represent the standardized residuals of individual participant loadings and nightmare index scores. Lines represent the linear line of best fit and the shaded bar represents the 95% confidence interval.

Given the potential visual representation of traumatic memories experienced in nightmares for individuals with PTSD [64], we completed additional analyses to directly assess if the SCN was related to nightmare experiences in the early aftermath of trauma (Fig. 2; Table S5). SCN loadings were linearly associated with 2-week nightmare severity [t(245) = 2.41, *β* = 0.15, 95% CI = [0.02, 0.28], *p*_corrected_ = 0.034] and intensity [t(245) = 2.64, *β* = 0.17, 95% CI = [0.04, 0.30], *p*_corrected_ = 0.027], but not frequency [t(245) = 1.81, *β* = 0.12, 95% CI = [− 0.01, 0.25], *p*_corrected_ = 0.071]. In line with the prior analyses, these data suggest that structural covariance of the VVS may be particularly related to traumatic visual memory encoding, retrieval, and expression.

### Structural covariance of the ventral visual stream moderates amygdala-hippocampal to inferior temporal gyrus connectivity

**Fig. 3:**
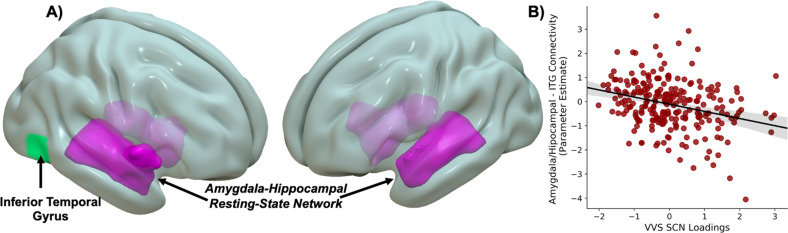
Ventral visual stream structural covariance strength is associated with arousal network connectivity to inferior temporal gyrus. We observed that strength of a structural covariance network of the ventral visual stream modulated connectivity between an amygdala-hippocampal functional covariance network and inferior temporal gyrus (**A**) such that greater structural covariance network loadings were associated with negative connectivity (**B**). Scatter plots are full plots where dots represent individual participant linked independent components analysis component loadings and parameter estimates of amygdala/hippocampal to inferior temporal gyrus connectivity. Lines represent the linear line of best fit and the shaded bar represents the 95% confidence interval.

Greater loadings of the VVS SCN were negatively related to resting-state functional connectivity between an amygdala-hippocampal RSN and the inferior temporal gyrus, a component of the VVS [Z_Peak_ = −3.87, *p*_corrected_ < 0.02, *k* = 61 (488 mm^3^), (XYZ = 52,−65,−7)] (Fig. 3). No other significant associations between VVS SCN loadings and resting-state connectivity were observed, even for RSNs that overlapped the VVS. No significant effects of VVS SCN loadings on amygdala reactivity to threat (fearful – neutral) were observed. These findings suggest structural covariance of the VVS impacts connectivity between the arousal network and components of the VVS.

### Future ventral visual stream structural covariance and posttraumatic stress symptoms

Prior to analysis of the 6-month imaging data, we assessed if the VVS SCN loadings at 2 weeks were related to participants’ 6-month PTSD symptoms. The regression models included linear and quadratic terms for 6-month PTSD symptoms, and covariates for scanner, age, and participant sex assigned at birth. A separate model also included additional covariates for prior trauma history and PTSD symptoms (i.e., prior to admission to the ED). No significant associations between the 2-week VVS SCN loadings and 6-month PTSD symptoms were observed (all *p* > 0.05).

**Fig. 4:**
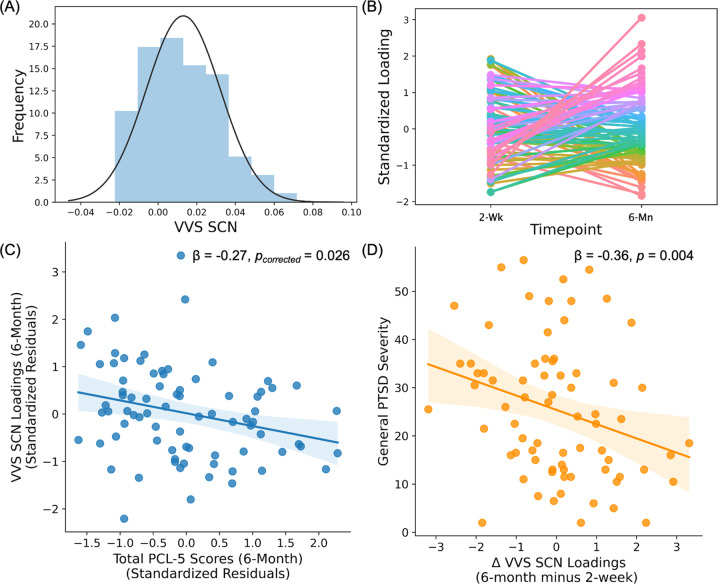
Stability of ventral visual stream structural covariance network over time. Similar to structural covariance network (SCN) loadings at 2 weeks, the 6-month loadings on component 21 were largely normally distributed (**A**). Individuals showed variability in component loadings between timepoint 1 (i.e., 2 weeks) and timepoint 2 (i.e., 6 months) post-trauma (**B**). The component loadings at 6 months were related to total scores on the PTSD Checklist for DSM-5 (PCL-5) at 6 months in models described in the “Methods” section (**C**). Participants who showed an overall decrease in SCN loadings over time showed greater PTSD symptom severity over time than those with increased SCN loadings (**D**). Scatter plots are partial plots where dots represent the standardized residuals of individual participant loadings and PCL-5 scores. Lines represent the linear line of best fit and the shaded bar represents the 95% confidence interval.

The SCN for the VVS was then projected onto participants’ 6-month MRI data to assess relationship between VVS SCN loadings and PTSD symptoms over time (Fig. 4; Table S6). In contrast to the positive association identified with 2-week loadings, VVS SCN loadings at 6 months were negatively associated with PTSD symptoms at 2 weeks [t(68) = −2.45, *β* = −0.27, 95% CI = [ − 0.45, −0.09], *p*_corrected_ = 0.034], however, the association was not significant when covarying for prior trauma history and pre-trauma PTSD symptoms [t(32) = −0.58, *β* = −0.12, 95% CI = [−0.62, 0.38], *p*_corrected_ = 0.564]. Further, VVS SCN loadings at 6 months were negatively associated with PTSD symptoms at 6 months [t(72) = −2.53, *β* = −0.27, 95% CI = [−0.45, −0.09], *p*_corrected_ = 0.026]. The effect was not significant when covarying for prior trauma history and pre-trauma PTSD symptoms [t(35) = −1.23, *β* = −0.26, 95% CI = [−0.73, 0.25], *p*_corrected_ = 0.229]. We also completed analyses to assess if there was a relationship between SCN loadings and individual PTSD symptom dimensions (indexed by PCL-5 subscale scores). At 6 months, SCN loadings were negatively associated with 6-month negative cognition and mood symptoms [t(72) = −2.40, *β* = −0.26, 95% CI = [−0.48, −0.07], *p* = 0.019], but not intrusion, avoidance, or arousal symptoms (all *p* > 0.05). Comparison of *β*-values revealed the association between the SCN loadings and negative alterations in cognition and mood symptoms was significantly different compared to the association with intrusion (Z-value = 2.36, *p* = 0.018), avoidance (Z-value = 2.23, 0.026), and arousal (Z-value = 2.13, *p* = 0.034) subscales. These findings suggest that, while greater structural covariance of the VVS facilitates encoding of traumatic memories acutely after trauma, decreased structural covariance over time contributes to negative trauma-related thoughts and feelings.

Together, the prior findings suggested that individuals at risk for greater PTSD symptoms showed relatively high structural integrity within the VVS early posttrauma, but also showed lower VVS integrity in the future. We completed a follow-up RM-ANCOVA to directly investigate associations between change over time in the VVS and PTSD symptoms. Although we did not observe a significant time by change in VVS SCN loadings interaction on PTSD symptoms [F(1,66) = 0.51, *p* = 0.480], the RM-ANCOVA revealed a significant effect of change in VVS SCN loadings on PTSD symptoms [F(1,66) = 8.82, *p* = 0.004]. Follow-up linear regressions revealed that decreased loadings over time were associated with greater PTSD symptoms [t(66) = −2.97, *β* = −0.36, 95% CI = [ − 0.60, −0.12], *p* = 0.004]. Of note, a follow-up paired-samples *t*-test of 2-week versus 6-month VVS SCN loadings did not reveal a significant difference [t(82) = 0.28, *p* = 0.781]. These data indicate individuals with high structural integrity within the ventral visual system in the early week post-trauma, and who lose this integrity over time, are most at risk for persistent PTSD-related dysfunction.

## Discussion

Multivariate MRI approaches hold promise for identifying neural signatures of susceptibility to trauma and stress-related disorders. However, limited work has investigated potential multivariate neural signatures of visual circuitry structure in recently traumatized individuals. In the current report, representing one of the largest prospective and longitudinal neuroimaging studies of trauma survivors, we identified a multimodal structural covariance network (SCN) of the ventral visual stream that is spatially consistent with our prior observations [33]. Loadings on the ventral visual stream SCN, 2 weeks post trauma exposure, were positively associated with 2-week PTSD symptoms. The SCN was also associated with both 2-week intrusion and nightmare symptoms. Further, loadings on the SCN varied with connectivity between the amygdala/hippocampus and inferior temporal gyrus which is part of the VVS. Interestingly, estimated loadings of the ventral visual stream SCN at 6 months were negatively associated with 6-month PTSD symptoms and individuals who showed decreased strength of the VVS SCN over time generally had higher PTSD symptoms. These findings provide multidimensional evidence of a role for the structure of the ventral visual stream in both the development and course of PTSD symptoms.

Multimodal data fusion via linked independent components analysis revealed an SCN that reflected white matter microstructure and gray matter morphology of the visual cortex, inferior fronto-occipital fasciculus, inferior temporal gyrus, and anterior temporal lobe (i.e., the ventral visual stream). The ventral visual stream is intimately involved in recognition of objects, their properties, and their representative meanings [12, 13] and is critical for processing of affective visual stimuli [9, 65, 66]. Importantly, the ventral visual stream may also be directly involved in memory retrieval processes for high arousal, threatening stimuli [67, 68]. In the present study, loadings on the ventral visual stream SCN were positively associated with acute (i.e., 2 weeks) PTSD symptoms. Given the positive loadings on the SCN reflected greater gray matter volume and pial surface area of visual stream regions, and the resulting positive association between SCN loadings and PTSD symptoms, it may be that greater structural integrity of the ventral visual stream in the early aftermath of trauma contributes to greater attention and reactivity to potentially threat-related visual stimuli. In turn, the network may facilitate encoding or consolidation of a threat-relevant visual memory. Enhanced neural ability to form strong visual memories may lead to stronger and more enduring trauma-related memories that ultimately contribute to the enhanced PTSD symptoms observed in the sample. Of note, our follow-up analyses revealed 2-week associations with the ventral visual stream SCN were more strongly related to intrusion symptoms consistent with our prior findings [33]. Thus, as opposed to increased consolidation, it may be that greater integrity of the network facilitates enhanced retrieval of the trauma-related threat memory and efficient reactivation of visual components of the trauma memory thereby exacerbating PTSD symptoms. The present findings cannot dissociate either the potential for enhanced consolidation or enhanced retrieval, nor can they rule out that both processes are acting in tandem in those with greater structural covariance of the ventral visual stream. We note, though, a possibility that the present results are unrelated to threat-processing and reflect a more domain-general process such that greater covariance could contribute to appetitive or highly salient memories regardless of valence. Future work should thus investigate threat and non-threat processing of the ventral visual stream in trauma-exposed individuals to better understand how the function of this circuitry is linked to PTSD.

Individual variability in the ventral visual stream SCN was positively associated with nightmare intensity. Though often overlooked, sleep disturbances are frequent and distressing consequences for trauma victims [8, 69–71]. Nightmare experiences are particularly damaging as they can contribute to negative emotional states and maladaptive behaviors in trauma victims [72–74]. It is possible, given the role of the ventral visual stream in emotion and determining representational meaning, that the observed association may be related to consolidation of trauma-related memories that may occur during sleep in line with our hypothesis above [75]. Individuals susceptible to posttraumatic nightmares may also be susceptible to more enduring but generalizable visual trauma memories which are in turn facilitated by the greater structural integrity of the ventral visual stream. However, it is not necessarily the case that the nightmares experienced by participants here are directly related to their traumatic event. Future work is needed to fully understand the relationship between posttraumatic nightmares and visual processing circuitry.

Loading strength of the ventral visual stream SCN was associated with amygdala-hippocampal connectivity to the inferior temporal gyrus (ITG). We recently demonstrated that “arousal network” to dorsolateral PFC as well as default mode to ITG connectivity was related to 3-month PTSD symptoms within the AURORA study [22]. The ITG cluster we observed in the present report showed high spatial overlap to that in the previous report with a similar central locus of the effect. The ITG is part of the anterior ventral visual stream and may support both high-level visual modeling and threat-specific processing as part of the ventral visual stream [76, 77]. In the present sample, greater structural covariance was associated with negative connectivity between the arousal network and ITG. The findings suggest that, in individuals with higher structural covariance of the ventral visual stream, arousing or salient contexts of stimuli are not properly integrated during encoding or retrieval of representative visual information regarding trauma cues. The suggested dysfunctional affective-visual encoding process may, in turn, be related to generalization of threat memories that characterize PTSD. This speculative process fits with the additional finding that greater loadings on the ventral visual stream SCN were associated with greater expression of acute PTSD symptoms. It may thus be somewhat surprising, given this interpretation, that no association was observed between SCN loadings and neural reactivity to threat indexed via a fearful face viewing task. The threat reactivity task has been used in several studies and shows pronounced activation of the amygdala and visual cortex, and it is associated with both acute and chronic posttraumatic dysfunction [20, 23, 36]. However, it is important to note that the present task involved passive viewing of stimuli without a requirement for participants to encode stimulus information such as valence. Given the previously described associations with intrusion symptoms, it may be that tasks that more explicitly involve emotional memory processes (e.g., Pavlovian threat conditioning) may show more robust associations with the ventral visual stream SCN. Together, these data suggest that amygdala-hippocampal connectivity with the posterior ventral visual stream may facilitate encoding of threat-relevant visual cues that ultimately contributes to PTSD susceptibility.

Of note, associations between ventral visual stream SCN loadings and PTSD symptoms were not stable between the 2-week and 6-month assessment timepoints. Thus, it may be that trauma-exposed participants’ ventral visual stream covariance represents a brain state that is unique in the immediate aftermath of, but changes over the 6 months following, trauma. Although 2-week ventral visual stream SCN loadings were positively associated with 2-week PTSD symptoms, we found ventral visual stream SCN loadings at 6 months were negatively associated with 6-month PTSD symptoms. Relatedly, individuals who showed decreases in SCN loadings over time also showed greater average PTSD symptoms at 6 months. The association between SCN loadings and acute posttraumatic symptoms was also strengthened when accounting for prior trauma and PTSD symptoms indicating a potential specificity of ventral visual stream integrity to stress reactivity in the early aftermath of trauma. These findings suggest that PTSD symptoms are associated with initially high, but then weakened, structural integrity of the ventral visual stream.

One way to conceptualize the present data is within a framework of delayed excitatory neurotoxicity effects of traumatic stress on brain structure. Acute stressors lead to selective neurogenesis and synaptic strengthening of circuits critical for formation of threat-relevant memories in animal models [78–80]. In humans, acute stress facilitates threat learning but contributes to overgeneralization which is further observed after trauma and in those with PTSD [81–83]. Threat-memory formation is highly glutamate-dependent, particularly within prefrontal-hippocampal circuitry, and dysregulated glutamatergic activity can lead to neural cell death and diminished gray matter volume [79, 84–86]. Thus, although acute stress can facilitate threat learning, supported by greater integrity of associated neural circuitry, chronic stress may lead to degeneration of the circuitry and contribute to delayed threat processing dysfunction. It may be that the chronic stress and potentially chronic activity of the ventral visual stream some individuals may experience following acute trauma have a deleterious effect on structural integrity of the visual pathway contributing to the differential associations. An interesting hypothesis for future testing relates to whether this apparent decreased visual stream structural integrity may contribute to generalization of visual threat cues seen in chronic PTSD. This framework may also help to explain dissociative findings in samples of recent trauma victims that show greater prefrontal glutamate/glutamine concentrations and null/mixed associations between brain volume and PTSD [30, 87, 88] compared to chronic PTSD samples [89–91].

Several limitations should be considered when interpreting the present findings. The present approach required participants to have complete MRI data across a number of features which reduced our sample size. It is important to note that participants who completed all MRI scans and had higher quality data may be phenotypically different from those who did not and these factors may be relevant for PTSD symptomatology. Thus, it is unclear if these data are generalizable to participants who drop out of research studies—who may in fact be most at risk for developing PTSD after trauma. It is also worth noting that, although we observed unique associations between VVS SCN loadings and specific PTSD symptom dimensions at 2 weeks and 6 months, such symptom dimensions are highly correlated with one another. Although we completed additional analyses with nightmare symptoms, additional data that assess psychological processes related to specific PTSD symptom dimensions (e.g., sensory sensitivity, memory consolidation strength, physiological reactivity) collected concurrently with MRI data may provide further support for the present findings. Another limitation is the lack of a non-trauma-exposed sample. Inclusion of a non-trauma sample is difficult given the preponderance of trauma in the U.S. (thereby questioning the ecological validity of the term “non-trauma”) and such a sample was excluded from AURORA due to feasibility constraints. Regardless, it remains unclear if trauma exposure itself may modulate the VVS SCN or if these would be observed in a non-trauma sample. Future work should investigate the occurrence and stability of a ventral visual stream SCN in non-trauma-exposed groups.

Our findings emphasize the often-overlooked role of sensory and particularly visual cortices in PTSD susceptibility after trauma. Further, the present data highlight potentially important relationship between the neural substrates of visual information processing and core threat neurocircuitry (e.g., amygdala/hippocampus) for understanding the development of PTSD. The current results also replicate prior findings in the largest-of-its-kind multisite, multimodal imaging study of recent trauma. Thus, modulation of visual neural circuitry after trauma opens new avenues for future research and potential neuromodulation techniques to reduce PTSD symptoms and nightmares in the aftermath of trauma [92–94]. Uncovering the nature of interactions between canonical threat and visual processing circuitry may provide the most effective avenue for the identification of robust and generalizable neural signatures of trauma and stress-related disorders.

## Supplementary information

Supplementary Material

## Data Availability

Data and/or research tools used in the preparation of this manuscript were obtained from the National Institute of Mental Health (NIMH) Data Archive (NDA). NDA is a collaborative informatics system created by the National Institutes of Health to provide a national resource to support and accelerate research in mental health. Dataset identifier(s): NIMH Data Archive Digital Object Identifier 10.15154/1526071. This manuscript reflects the views of the authors and may not reflect the opinions or views of the NIH or of the Submitters submitting original data to NDA.

## Code availability

Code for the present study was obtained as part of publicly available distributions of FMRIPREP (described in “Methods” and supplement), FSL, and AFNI. Commercial software was used in statistical analysis. Detailed code is available upon request.
