# Supplementary material for: Structural covariance of the ventral visual stream predicts posttraumatic intrusion and nightmare symptoms: a multivariate data fusion analysis
Source: Transl Psychiatry. 2022 Aug 8;12:321. doi: 10.1038/s41398-022-02085-8 (PMC9360028; doi:10.1038/s41398-022-02085-8)

**Supplementary Information:**

***Structural covariance of the ventral visual stream predicts posttraumatic intrusion and nightmare symptoms: a multivariate data fusion analysis.***

Supplementary Methods

Magnetic Resonance Imaging

Results included in this manuscript come from preprocessing performed using FMRIPREP version stable 1.2.2 [1, 2, RRID:SCR_016216], a Nipype [3, 4, RRID:SCR_002502] based tool. Each T1w (T1-weighted) volume was corrected for INU (intensity non-uniformity) using N4BiasFieldCorrection v2.1.0 [5] and skull-stripped using antsBrainExtraction.sh v2.1.0 (using the OASIS template). Brain surfaces were reconstructed using recon-all from FreeSurfer v6.0.1 [6, RRID:SCR_001847], and the brain mask estimated previously was refined with a custom variation of the method to reconcile ANTs-derived and FreeSurfer-derived segmentations of the cortical gray-matter of Mindboggle [21, RRID:SCR_002438]. Spatial normalization to the ICBM 152 Nonlinear Asymmetrical template version 2009c [7, RRID:SCR_008796] was performed through nonlinear registration with the antsRegistration tool of ANTs v2.1.0 [8, RRID:SCR_004757], using brain-extracted versions of both T1w volume and template. Brain tissue segmentation of cerebrospinal fluid (CSF), white-matter (WM) and gray-matter (GM) was performed on the brain-extracted T1w using fast [17] (FSL v5.0.9, RRID:SCR_002823). Functional data was slice time corrected using 3dTshift from AFNI v16.2.07 [11, RRID:SCR_005927] and motion corrected using mcflirt (FSL v5.0.9 [9]). This was followed by co-registration to the corresponding T1w using boundary-based registration [16] with six degrees of freedom, using bbregister (FreeSurfer v6.0.1). Motion correcting transformations, BOLD-to-T1w transformation and T1w-to-template (MNI) warp were concatenated and applied in a single step using antsApplyTransforms (ANTs v2.1.0) using Lanczos interpolation. Frame-wise displacement [19] was calculated for each functional run using the implementation of Nipype. ICA-based Automatic Removal Of Motion Artifacts (AROMA) was used to generate aggressive noise regressors as well as to create a variant of data that is non-aggressively denoised [20]. Many internal operations of FMRIPREP use Nilearn [22, RRID:SCR_001362], principally within the BOLD-processing workflow. For more details of the pipeline see <https://fmriprep.readthedocs.io/en/stable/workflows.html>.

MRI Quality Control Measures

For T1-weighted data, we assessed sitewise differences in MRI quality control metric in MRI-QC: coefficient of joint variation (CJV) of gray and white matter, median intensity nonuniformity (INU), and the signal-to-noise ratio (SNR). CJV is thought to reflect the presence of heavy head motion and artifacts where lower scores reflect better quality data. INU reflects non-anatomically variation in signal intensity across the volume. SNR is the signal to noise ratio of the entire T1-volume across tissue types. For DWI data, we assessed sitewise differences in MRI quality control metrics derived from initial processing [23]: temporal signal-to-noise ratio (TSNR), maximum outlier voxel intensity (OUTMAX), mean absolute motion (MEANABS), and maximum absolute motion (MAXABS). TSNR is the temporally averaged signal to noise ratio for each dataset. For functional data, we assessed sitewise differences in MRI quality control metrics in MRI-QC [23]: the AFNI Quality Index (AQI), FD, DVARs, and temporal-signal-to-noise ratio (TSNR). AQI is a general and crude screening tool for motion or scanner artifacts in 4D datasets. AQI is calculated as an average of 1 minus the Spearman rank correlation coefficient for every volume to the median volume in the dataset. Framewise displacement is an estimation of head-movement across the dataset [24]. DVARs is calculated as the derivative of the root-mean-square variance over dataset voxels [24]. Site-wise statistics are provided in the supplementary results and Figure S2.

Supplementary Results

*Sitewise differences in quality assurance metrics*

*T1 Metrics*

We observed significant site differences in CJV [F(4,273) = 46.52, p < 0.001], median INU [F(4,273) = 6.11, p < 0.001], and SNR [F(4,273) = 18.55, p < 0.001] values. Posthoc t-tests with Tukey correction revealed Site 1 had higher CJV than Site 2, Site 3, and Site 4. Site 2 had high CJV than Site 3 and Site 4, but lower CJV than Site 5. Site 3 had low CJV than Site 4 and Site 5, and Site 4 had lower CJV than Site 5 (all p<0.003). Site 4 had lower median INU than Site 1 (p = 0.023), Site 2 (p = 0.001), and Site 3 (p < 0.001). Site 3 had greater SNR than Site 1, Site 2, Site 4, and Site 5 (all p < 0.001). Site 1 also had lower SNR than Site 5 (p = 0.021).

*DWI Metrics*

No significant main effects of site were observed on OUTMAX [F(4,273) = 2.36, p = 0.054], MEANABS [F(4,273) = 1.10, p = 0.357], or MAXABS [F(4,273) = 1.29, p = 0.275]. A significant effect of site was observed on TSNR [F(4,273) = 282.65, p < 0.001]. Posthoc t-tests with Tukey correction revealed significant differences between Site 1 had lower TSNR than Site 3 (p<0.001) and Site 5 (p<0.001). Site 2 had significantly lower TSNR than Site 3 and Site 5 (all p < 0.001). Site 3 and Site 5 had greater TSNR than Site 4 (all p<0.001).

*Resting-state fMRI*

We observed significant differences across scan sites in AQI [F(4,273) = 48.06, p <0.001], DVARS [F(4,273) = 2.84, p = 0.005], and TSNR [F(4,273) = 32.68, p < 0.001]. AQI was greater in the Site 1 site compared to Site 3 and Site 5 (all p < 0.001). AQI in the Site 2 site was greater than Site 3 and Site 5 (all p < 0.001). Site 4 had greater AQI than Site 3 and Site 5 (all p < 0.001). No pairwise comparisons survived Tukey correction for DVARS. Site 3 and Site 5 had greater TSNR than Site 1, Site 2, and Site 4 (all p < 0.001). Site 2 had greater TSNR than Site 4 (p = 0.036). No sitewise differences were observed in FD [F(4,273) = 0.02 p = 0.939].

*Task fMRI*

We observed significant differences in AQI F(4,273) = 36.17, p <0.001], DVARS F(4,273) = 6.37, p <0.001], FD F(4,273) = 2.82, p = 0.025], and TSNR F(4,273) = 30.76, p <0.001]. Site 1 had greater AQI than all other sites (all p < 0.05). Site 2 and Site 4 had greater AQI than Site 3 and Site 5 (all p < 0.001). Site 3 had lower DVARS than Site 1 and Site 4 (all p < 0.05). Site 2 had greater DVARS than Site 3 (p = 0.002). Differences in FD were driven by greater FD in Site 1 compared to Site 2, Site 3, and Site 4 (all p < 0.05). Site 3 and Site 4 had greater TSNR than Site 1, Site 2, and Site 4 (all p < 0.05).

Supplementary References

1. Esteban O, Markiewicz CJ, Blair RW, Moodie CA, Isik AI, Erramuzpe A, Kent JD, Goncalves M, DuPre E, Snyder M, Oya H, Ghosh SS, Wright J, Durnez J, Poldrack RA, Gorgolewski KJ. fMRIPrep: a robust preprocessing pipeline for functional MRI. Nat Meth. 2018; doi:[10.1038/s41592-018-0235-4](https://doi.org/10.1038/s41592-018-0235-4)

2. fMRIPrep Available from: [10.5281/zenodo.852659](https://doi.org/10.5281/zenodo.852659).

3. Gorgolewski K, Burns CD, Madison C, Clark D, Halchenko YO, Waskom ML, Ghosh SS. Nipype: a flexible, lightweight and extensible neuroimaging data processing framework in python. Front Neuroinform. 2011 Aug 22;5(August):13. doi:[10.3389/fninf.2011.00013](https://doi.org/10.3389/fninf.2011.00013).

4. Gorgolewski KJ, Esteban O, Ellis DG, Notter MP, Ziegler E, Johnson H, Hamalainen C, Yvernault B, Burns C, Manhães-Savio A, Jarecka D, Markiewicz CJ, Salo T, Clark D, Waskom M, Wong J, Modat M, Dewey BE, Clark MG, Dayan M, Loney F, Madison C, Gramfort A, Keshavan A, Berleant S, Pinsard B, Goncalves M, Clark D, Cipollini B, Varoquaux G, Wassermann D, Rokem A, Halchenko YO, Forbes J, Moloney B, Malone IB, Hanke M, Mordom D, Buchanan C, Pauli WM, Huntenburg JM, Horea C, Schwartz Y, Tungaraza R, Iqbal S, Kleesiek J, Sikka S, Frohlich C, Kent J, Perez-Guevara M, Watanabe A, Welch D, Cumba C, Ginsburg D, Eshaghi A, Kastman E, Bougacha S, Blair R, Acland B, Gillman A, Schaefer A, Nichols BN, Giavasis S, Erickson D, Correa C, Ghayoor A, Küttner R, Haselgrove C, Zhou D, Craddock RC, Haehn D, Lampe L, Millman J, Lai J, Renfro M, Liu S, Stadler J, Glatard T, Kahn AE, Kong X-Z, Triplett W, Park A, McDermottroe C, Hallquist M, Poldrack R, Perkins LN, Noel M, Gerhard S, Salvatore J, Mertz F, Broderick W, Inati S, Hinds O, Brett M, Durnez J, Tambini A, Rothmei S, Andberg SK, Cooper G, Marina A, Mattfeld A, Urchs S, Sharp P, Matsubara K, Geisler D, Cheung B, Floren A, Nickson T, Pannetier N, Weinstein A, Dubois M, Arias J, Tarbert C, Schlamp K, Jordan K, Liem F, Saase V, Harms R, Khanuja R, Podranski K, Flandin G, Papadopoulos Orfanos D, Schwabacher I, McNamee D, Falkiewicz M, Pellman J, Linkersdörfer J, Varada J, Pérez-García F, Davison A, Shachnev D, Ghosh S. Nipype: a flexible, lightweight and extensible neuroimaging data processing framework in Python. 2017. doi:[10.5281/zenodo.581704](https://doi.org/10.5281/zenodo.581704).

5. Tustison NJ, Avants BB, Cook PA, Zheng Y, Egan A, Yushkevich PA, Gee JC. N4ITK: improved N3 bias correction. IEEE Trans Med Imaging. 2010 Jun;29(6):1310–20. doi:[10.1109/TMI.2010.2046908](https://doi.org/10.1109/TMI.2010.2046908).

6. Dale A, Fischl B, Sereno MI. Cortical Surface-Based Analysis: I. Segmentation and Surface Reconstruction. Neuroimage. 1999;9(2):179–94. doi:[10.1006/nimg.1998.0395](https://doi.org/10.1006/nimg.1998.0395).

7. Fonov VS, Evans AC, McKinstry RC, Almli CR, Collins DL. Unbiased nonlinear average age-appropriate brain templates from birth to adulthood. NeuroImage; Amsterdam. 2009 Jul 1;47:S102. doi:[10.1016/S1053-8119(09)70884-5](https://doi.org/10.1016/S1053-8119(09)70884-5).

8. Avants BB, Epstein CL, Grossman M, Gee JC. Symmetric diffeomorphic image registration with cross-correlation: evaluating automated labeling of elderly and neurodegenerative brain. Med Image Anal. 2008 Feb;12(1):26–41. doi:[10.1016/j.media.2007.06.004](https://doi.org/10.1016/j.media.2007.06.004).

9. Jenkinson M, Bannister P, Brady M, Smith S. Improved optimization for the robust and accurate linear registration and motion correction of brain images. Neuroimage. 2002 Oct;17(2):825–41. doi:[10.1006/nimg.2002.1132](https://doi.org/10.1006/nimg.2002.1132).

10. Andersson JLR, Skare S, Ashburner J. How to correct susceptibility distortions in spin-echo echo-planar images: application to diffusion tensor imaging. Neuroimage. 2003 Oct;20(2):870–88. doi:[10.1016/S1053-8119(03)00336-7](https://doi.org/10.1016/S1053-8119(03)00336-7).

11. Cox RW. AFNI: software for analysis and visualization of functional magnetic resonance neuroimages. Comput Biomed Res. 1996 Jun;29(3):162–73. doi:[10.1006/cbmr.1996.0014](https://doi.org/10.1006/cbmr.1996.0014).

12. Jenkinson M. Fast, automated, N-dimensional phase-unwrapping algorithm. Magn Reson Med. 2003 Jan;49(1):193–7. doi:[10.1002/mrm.10354](https://doi.org/10.1002/mrm.10354).

13. Huntenburg JM. Evaluating nonlinear coregistration of BOLD EPI and T1w images. Freie Universität Berlin; 2014. Available from: <http://hdl.handle.net/11858/00-001M-0000-002B-1CB5-A>.

14. Wang S, Peterson DJ, Gatenby JC, Li W, Grabowski TJ, Madhyastha TM. Evaluation of Field Map and Nonlinear Registration Methods for Correction of Susceptibility Artifacts in Diffusion MRI. Front Neuroinform. 2017 [cited 2017 Feb 21];11. doi:[10.3389/fninf.2017.00017](https://doi.org/10.3389/fninf.2017.00017).

15. Treiber JM, White NS, Steed TC, Bartsch H, Holland D, Farid N, McDonald CR, Carter BS, Dale AM, Chen CC. Characterization and Correction of Geometric Distortions in 814 Diffusion Weighted Images. PLoS One. 2016 Mar 30;11(3):e0152472. doi:[10.1371/journal.pone.0152472](https://doi.org/10.1371/journal.pone.0152472).

16. Greve DN, Fischl B. Accurate and robust brain image alignment using boundary-based registration. Neuroimage. 2009 Oct;48(1):63–72. doi:[10.1016/j.neuroimage.2009.06.060](https://doi.org/10.1016/j.neuroimage.2009.06.060).

17. Zhang Y, Brady M, Smith S. Segmentation of brain MR images through a hidden Markov random field model and the expectation-maximization algorithm. IEEE Trans Med Imaging. 2001 Jan;20(1):45–57. doi:[10.1109/42.906424](https://doi.org/10.1109/42.906424).

18. Behzadi Y, Restom K, Liau J, Liu TT. A component based noise correction method (CompCor) for BOLD and perfusion based fMRI. Neuroimage. 2007 Aug 1;37(1):90–101. doi:[10.1016/j.neuroimage.2007.04.042](https://doi.org/10.1016/j.neuroimage.2007.04.042).

19. Power JD, Mitra A, Laumann TO, Snyder AZ, Schlaggar BL, Petersen SE. Methods to detect, characterize, and remove motion artifact in resting state fMRI. Neuroimage. 2013 Aug 29;84:320–41. doi:[10.1016/j.neuroimage.2013.08.048](https://doi.org/10.1016/j.neuroimage.2013.08.048).

20. Pruim RHR, Mennes M, van Rooij D, Llera A, Buitelaar JK, Beckmann CF. ICA-AROMA: A robust ICA-based strategy for removing motion artifacts from fMRI data. Neuroimage. 2015 May 15;112:267–77. doi:[10.1016/j.neuroimage.2015.02.064](https://doi.org/10.1016/j.neuroimage.2015.02.064).

21. Klein A, Ghosh SS, Bao FS, Giard J, Häme Y, Stavsky E, et al. Mindboggling morphometry of human brains. PLoS Comput Biol 13(2): e1005350. 2017. doi:[10.1371/journal.pcbi.1005350](https://doi.org/10.1371/journal.pcbi.1005350).

22. Abraham A, Pedregosa F, Eickenberg M, Gervais P, Mueller A, Kossaifi J, Gramfort A, Thirion B, Varoquaux G. Machine learning for neuroimaging with scikit-learn. Front in Neuroinf 8:14. 2014. doi:[10.3389/fninf.2014.00014](https://doi.org/10.3389/fninf.2014.00014).

23. Esteban, O., Birman, D., Schaer, M., Koyejo, O. O., Poldrack, R. A., & Gorgolewski, K. J. (2017). MRIQC: Advancing the automatic prediction of image quality in MRI from unseen sites. PloS One, 12(9), e0184661. https://doi.org/10.1371/journal.pone.0184661

24. Power, J. D., Barnes, K. A., Snyder, A. Z., Schlaggar, B. L., & Petersen, S. E. (2012). Spurious but systematic correlations in functional connectivity MRI networks arise from subject motion. NeuroImage, 59(3), 2142–2154. <https://doi.org/10.1016/j.neuroimage.2011.10.018>

Table S1. Broad-class trauma exposures in the present sample

| **Broad class trauma** | **Frequency** |
| --- | --- |
| Motor Vehicle Collision | 201 |
| Physical Assault | 31 |
| Sexual Assault | 3 |
| Fall >= 10 feet | 3 |
| Incident causing traumatic stress exposure to many people | 1 |
| Non-motorized collision | 11 |
| Fall < 10 feet | 11 |
| Burns | 1 |
| Animal-related trauma | 9 |
| Other | 7 |

Table S2. MRI sequences by site.

|  | Site1  Siemens TIM 3T Trio  (12 Channel Head Coil) | Site2  Siemens TIM 3T Trio  (12 Channel Head Coil) | Site3  Siemens MAGNETOM 3T Prisma  (20 Channel Head Coil) | Site4  Siemens 3T Verio  (12 Channel Head Coil) | Site5  Siemens MAGNETOM 3T Prisma  (20 Channel Head Coil) |
| --- | --- | --- | --- | --- | --- |
| Modality |  |  |  |  |  |
| T1-weighted | **TR** = 2530ms, **TEs** = 1.74/3.6/5.46/7.32ms, **TI** = 1260ms, **flip angle** = 7, **FOV** = 256mm, **slices** = 176, **Voxel size** = 1mm x 1mm x 1mm | **TR** = 2530ms, **TEs** = 1.74/3.6/5.46/7.32ms, **TI** = 1260ms, **flip angle** = 7, **FOV** = 256mm, **slices** = 176, **Voxel size** = 1mm x 1mm x 1mm | **TR** = 2300ms, **TE** = 2.96ms, **TI** = 900ms, **flip angle** = 9, **FOV** = 256mm, **slices** = 176, **Voxel size** = 1.2mm x 1.0mm x 12mm | **TR** = 2530ms, **TEs** = 1.74/3.65/5.51/7.72ms, **TI** = 1260ms, **flip angle** = 7, **FOV** = 256mm, **slices** = 176, **Voxel size** = 1mm x 1mm x 1mm | **TR** = 2300ms, **TE** = 2.98ms, **TI** = 900ms, **flip angle** = 9, **FOV** = 256mm, **slices** = 176, **Voxel size** = 1.2mm x 1.0mm x 12mm |
| Diffusion Weighted Imaging | **TR** = 7700ms, **TE** = 85ms, **FOV** = 212mm, **flip angle** = 90, **Volumes** = 71 (64 **b**=1000 s/mm^2,^ 7 b0), **PA-encoded**, **Voxel size** = 2mm x 2mm x 2mm | **TR** = 7700ms, **TE** = 85ms, **FOV** = 212mm, **flip angle** = 90, **Volumes** = 71 (64 **b**=1000 s/mm^2,^ 7 b0), **PA-encoded**, **Voxel size** = 2mm x 2mm x 2mm | **TR** = 7000ms, **TE** = 74ms, **FOV** = 212mm, **flip angle** = 90, **Volumes** = 71 (64 **b**=1000 s/mm^2,^ 7 b0), **PA-encoded**, **Voxel size** = 2mm x 2mm x 2mm | **TR** = 12000ms, **TE** = 85ms, **FOV** = 212mm, **flip angle** = 90, **Volumes** = 71 (64 **b**=1000 s/mm^2,^ 7 b0), **PA-encoded**, **Voxel size** = 2mm x 2mm x 2mm | **TR** = 7700ms, **TE** = 67ms, **FOV** = 212mm, **flip angle** = 90, **Volumes** = 71 (64 **b**=1000 s/mm^2,^ 7 b0), **PA-encoded**, **Voxel size** = 2mm x 2mm x 2mm |
| Functional MRI | **TR** = 2360ms, **TE** = 30ms, **flip angle** = 70, **FOV** = 212mm, **slices** = 44, **Voxel size** = 3mm x 2.72mm x 2.72mm, 0.5 mm gap | **TR** = 2360ms, **TE** = 30ms, **flip angle** = 70, **FOV** = 212mm, **slices** = 44, **Voxel size** = 3mm x 3mm x 3mm, 0.5 mm gap | **TR** = 2360ms, **TE** = 29ms, **flip angle** = 70, **FOV** = 212mm, **slices** = 44, **Voxel size** = 3mm x 2.72mm x 2.72mm, 0.5 mm gap | **TR** = 2360ms, **TE** = 30ms, **flip angle** = 70, **FOV** = 212mm, **slices** = 42, **Voxel size** = 3mm x 2.72mm x 2.72mm, 0.5 mm gap | **TR** = 2360ms, **TE** = 29ms, **flip angle** = 90, **FOV** = 210mm, **slices** = 44, **Voxel size** = 3mm x 3mm x 2.5mm, 0.5 mm gap |

Table S3. Full model of ventral visual stream associations with 2-week PCL-5 scores.

|  | Model A | Model B |
| --- | --- | --- |
| F-Statistic  (p-value) | 0.83  (0.578) | 1.11  (0.357) |
| R^2^ | 0.03 | 0.08 |
| Variable | β (p-value) | β (p-value) |
| Age | -0.04 (0.535) | 0.04 (0.663) |
| Sex Assigned at Birth | 0.17 (0.346) | -0.26 (0.596) |
| Scanner1 | 0.11 (0.509) | -0.29 (0.546) |
| Scanner2 | 0.11 (0.508) | -0.25 (0.556) |
| Scanner3 | -0.04 (0.743) | -0.23 (0.375) |
| Scanner4 | -0.01 (0.864) | 0.03 (0.743) |
| PCL-5 (Linear) | 0.123 (0.040)* | 0.22 (0.025)* |
| PCL-5 (Quadratic) | -0.02 (0.797) | -0.10 (0.32) |
| PCL-5 (Prior) | . | 0.06 (0.575) |
| LEC-5 | . | 0.08 (0.36) |

Note: PCL-5 = PTSD Checklist for DSM-5; LEC-5 = Life Events Checklist for DSM-5; *p<0.05, 1-tailed hypothesis testing

Table S4. Full model of ventral visual stream associations with 2-week PROMIS Depression scores.

|  | Model A | Model B |
| --- | --- | --- |
| F-Statistic  (p-value) | 0.37  (0.936) | 0.729  (0.697) |
| R^2^ | 0.01 | 0.04 |
| Variable | β (p-value) | β (p-value) |
| Age | -0.01 (0.851) | 0.03 (0.685) |
| Sex Assigned at Birth | 0.00 (0.995) | 0.02 (0.758) |
| Scanner1 | 0.17 (0.352) | 0.26 (0.467) |
| Scanner2 | 0.13 (0.440) | 0.20 (0.544) |
| Scanner3 | 0.10 (0.532) | 0.16 (0.609) |
| Scanner4 | -0.01 (0.894) | 0.02 (0.921) |
| PROMIS Depression (Linear) | 0.00 (0.963) | -0.04 (0.630) |
| PROMIS Depression (Quadratic) | -0.02 (0.725) | -0.03 (0.731) |
| PROMIS Depression (Prior) | . | 0.06 (0.450) |
| LEC-5 | . | 0.12 (0.089) |

Note: PRMOIS = Patient-Reported Outcomes Measurement Information System; LEC-5 = Life Events Checklist for DSM-5.

Table S5. Full model of ventral visual stream associations with nightmare symptoms

|  | Frequency | Intensity | Severity |
| --- | --- | --- | --- |
| F-Statistic  (p-value) | 0.90 (0.509) | 1.43 (0.194) | 1.26 (0.27) |
| R^2^ | 0.03 | 0.04 | 0.04 |
| Variable | β (p-value) | β (p-value) | β (p-value) |
| Age | -0.02 (0.753) | -0.02 (0.803) | -0.02 (0.76) |
| Sex Assigned at Birth | 0.16 (0.387) | 0.14 (0.439) | 0.15 (0.421) |
| Scanner1 | 0.13 (0.457) | 0.11 (0.51) | 0.12 (0.492) |
| Scanner2 | 0.10 (0.553) | 0.09 (0.574) | 0.09 (0.565) |
| Scanner3 | -0.02 (0.832) | -0.03 (0.737) | -0.03 (0.771) |
| Scanner4 | -0.02 (0.794) | -0.02 (0.728) | -0.02 (0.736) |
| Nightmare Symptoms | 0.12 (0.071) | 0.17 (0.009)** | 0.15 (0.017)* |

Note: *p<0.05, **p<0.05 after Bonferroni correction.

Table S6. Full model of 6-month ventral visual stream associations with 6-month PCL-5 scores.

|  | Model A | Model B |
| --- | --- | --- |
| F-Statistic (p-value) | 3.32 (0.006) | 1.09 (0.394) |
| R^2^ | 0.22 | 0.20 |
| Variable | β (p-value) | β (p-value) |
| Age | 0.31 (0.004) | 0.25 (0.136) |
| Sex Assigned at Birth | 0.18 (0.133) | 0.15 (0.434) |
| Scanner2 | 0.18 (0.123) | 0.07 (0.7) |
| Scanner3 | 0.14 (0.193) | 0.16 (0.327) |
| Scanner4 | 0.01 (0.902) | -0.05 (0.779) |
| PCL-5 (6-months) | -0.27 (0.013) | -0.26 (0.229) |
| PCL-5 (Prior) | . | 0.16 (0.427) |
| LEC-5 | . | -0.20 (0.261) |

Note: PCL-5 = PTSD Checklist for DSM-5; LEC-5 = Life Events Checklist for DSM-5

Figure S1. **Schematic of data processing for multimodal fusion and statistical analysis.** T1-weighted, DWI, and functional MRI data were processed using pipelines reported in the main text. T1-weighted data was processed using VBM and FreeSurfer pipelines, while DWI data was processed using FSL and custom code. Features derived from these pipelines were including in LICA to identify structural covariance networks and we selected one component for statistical analysis. In parallel, task-fMRI and resting-state fMRI data were processed in FMRIPREP with further modality specific processing to complete 1^st^-level models (task-fMRI data) and participant-specific resting-state network maps for further statistical analysis with the participant loadings on the structural covariance network of interest.


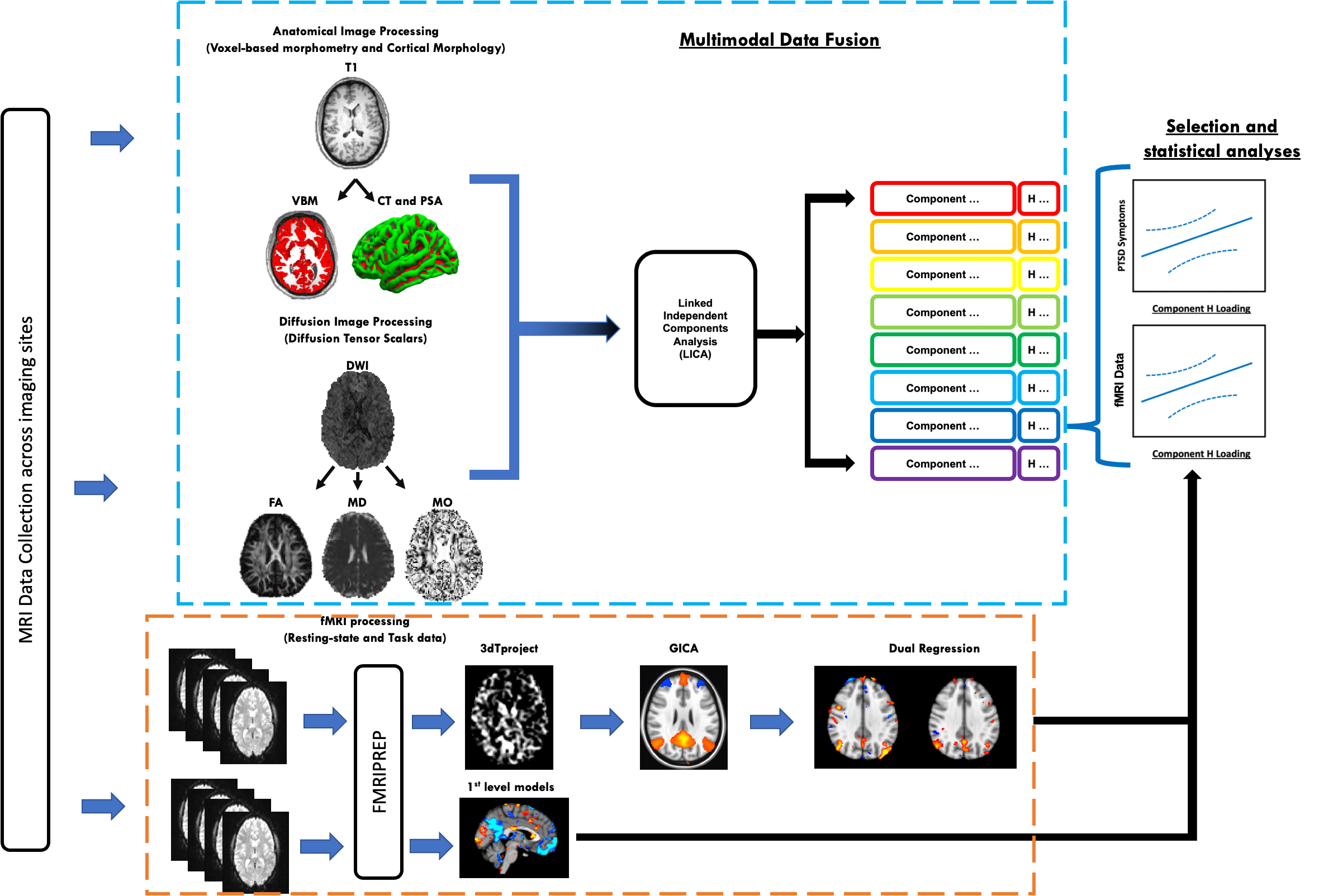


Figure S2. **Resting-state networks considered in analyses.** Components from MELODIC were selected for voxelwise analysis of network-to-node connectivity with ventral visual stream structural covariance network loadings. We initially selected two RSNs, the default mode network and an amygdala-hippocampal arousal network (noted by *s), though also considered 6 additional visual/arousal components that overlapped our *a priori* regions of interest (i.e., the ventral visual stream structural covariance network).

**
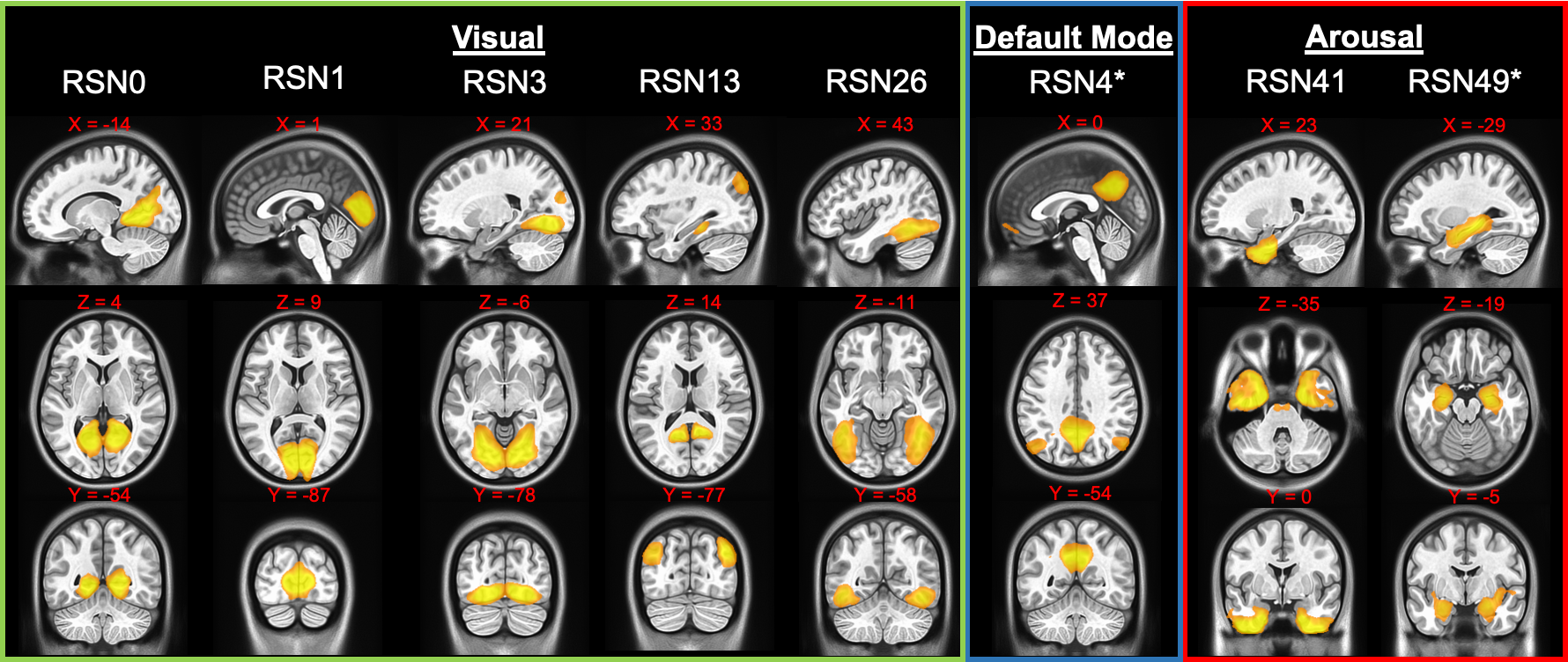
**

Figure S3. **Comparison of MRI quality control metrics for each site by modality.** For completeness, we compared MRI quality control metrics for each modality including (A) T1-weighted anatomical images (Signal-to-Noise Ratio, Intensity Non-Uniformity, Coeffecient of Join Variation), (B) Diffusion-weighted images (Temporal Signal-to-Noise Ratio, Outlier Maximum Voxel Intensity, Mean Absolute Motion, Maximum Absolute Motion), and both (C) resting state and (D) task functional MRI data (Temporal Signal-to-Noise Ratio, AFNI Quality Index, DVARS, Mean Framewise Displacement).


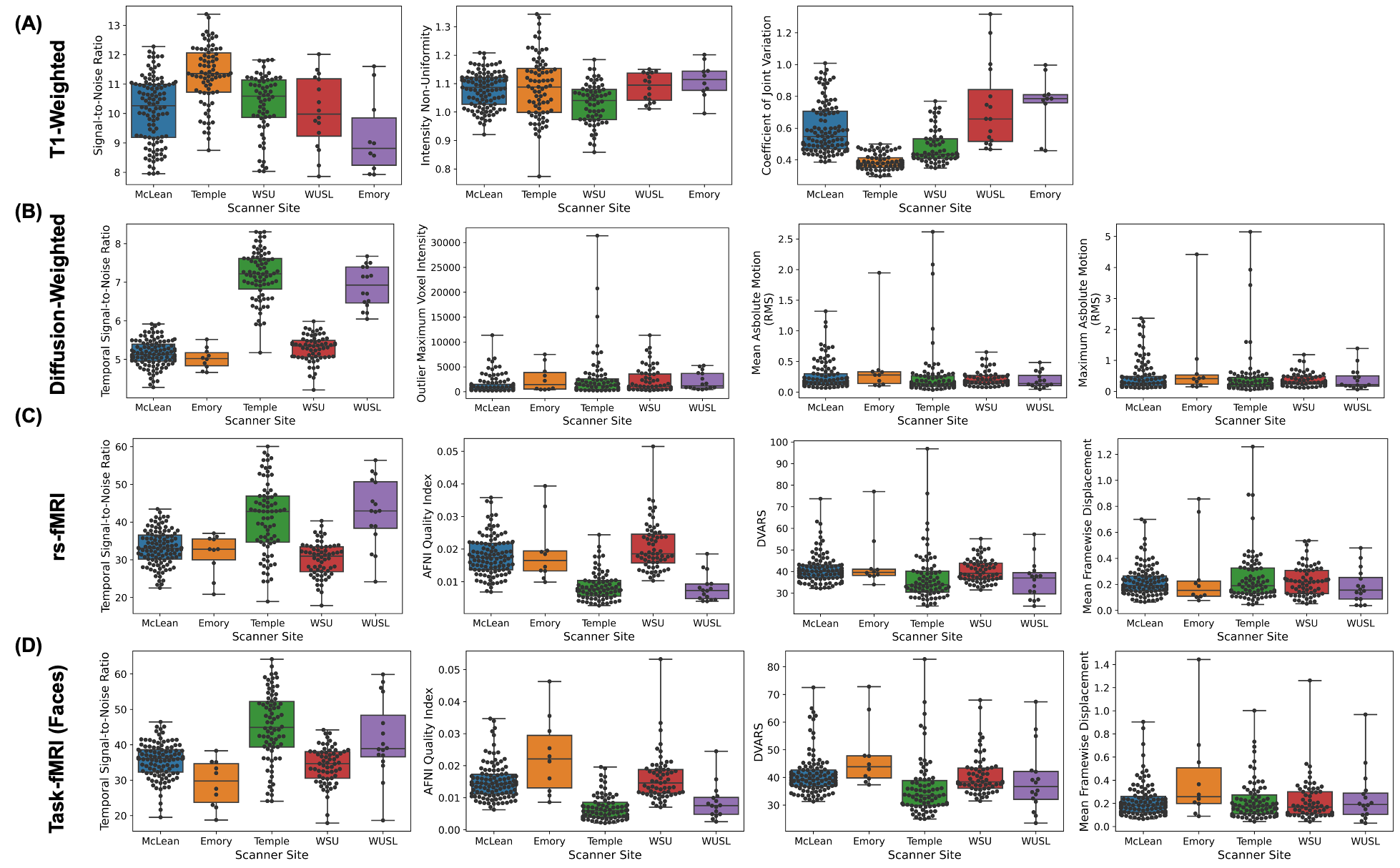

Supplement: Supplementary file 1 — Supplementary Material [file 41398_2022_2085_MOESM1_ESM.docx]
